# Analysis of the DNA methylation of the *H19* gene in human bladder cancer

**DOI:** 10.1186/1753-6561-7-S2-P47

**Published:** 2013-04-04

**Authors:** Mariana B Reis, Priscila M Ramos, João LV Camargo, Cláudia A Rainho

**Affiliations:** 1 Department of Genetics, Biosciences Institute, UNESP, Sao Paulo State University, Botucatu, SP, Brazil; 2 Department of Pathology, Botucatu Medical School, UNESP, Sao Paulo State University, Botucatu, SP, Brazil

## Background

*H19* is a paternally imprinted gene located at 11p15.5, which encodes a non-coding transcript. Although the role of genomic imprinting in bladder cancer is not well understood, previous studies have described *H19* over-expression in these tumors. It is well established that a Differentially Methylated Region (DMR) regulates its maternal monoallelic expression by acting as an insulator that precludes the binding of the transcriptional factor CTCF in the paternal allele.

## Materials and methods

DNA methylation *status* of two distinct regions of the *H19* gene was evaluated: the sixth CTCF-binding site located in the DMR (qMSP, Quantitative Real Time Methylation Specific Polymerase Chain Reaction) and the promoter-associated CpG island (MS-HRM, Methylation-Sensitive High Resolution Melting analysis) in a total of 48 tumoral samples (37 of them matched with normal adjacent tissue).

## Results

Using a pool of blood samples obtained from healthy young adults as reference to the normal imprinting, higher methylation levels of the CTCF-binding site was detected in bladder tumors compared to the normal adjacent tissue (*p*=*0.0031*). Gains of methylation were more frequently detected in non-invasive (*p*=*0.0425*) and non-recurrent (*p*=*0.0399*) papillary bladder tumors. While DNA methylation levels of *H19* promoter region varied from 10 to 50% in normal adjacent bladder tissues, tumoral samples showed greater variation (10 to 100% of methylation). Heterogeneous patterns of CpG methylation were also detected in nine tumoral samples.

## Conclusion(s)

Our data suggest that aberrant DNA methylation is an epigenetic change potentially associated with loss of imprinting of the *H19* gene in bladder cancer.

## Financial Support

FAPESP, CAPES.

